# The Perils of Misinterpreting and Misusing “Publication Bias” in Meta-analyses: An Education Review on Funnel Plot-Based Methods

**DOI:** 10.1007/s40279-023-01927-9

**Published:** 2023-09-08

**Authors:** José Afonso, Rodrigo Ramirez-Campillo, Filipe Manuel Clemente, Fionn Cléirigh Büttner, Renato Andrade

**Affiliations:** 1 Faculty of Sport, Centre of Research, Education, Innovation, and Intervention in Sport (CIFI2D), University of Porto, Porto, Portugal; 2 Exercise and Rehabilitation Sciences Institute, Faculty of Rehabilitation Sciences, School of Physical Therapy, Universidad Andres Bello, Santiago, Chile; 3 Escola Superior de Desporto e Lazer, Instituto Politécnico de Viana do Castelo, Rua Escola Industrial Comercial de Nun’Álvares, 4900-347 Viana do Castelo, Portugal; 4 Instituto de Telecomunicações, Delegação da Covilhã, Covilhã, Portugal; 5 Research Center in Sports Performance, Recreation, Innovation and Technology (SPRINT), 4960-320 Melgaço, Portugal; 6 Pragmatic Clinical Trials Unit, Centre for Evaluation and Methods, Wolfson Centre of Population Health, Queen Mary University of London, London, UK; 7 Department of Medical Statistics, London School of Hygiene and Tropical Medicine, London, UK; 8 Porto Biomechanics Laboratory (LABIOMEP), University of Porto, Porto, Portugal; 9 Clínica Espregueira, FIFA Medical Centre of Excellence, Porto, Portugal; 10 Dom Henrique Research Centre, Porto, Portugal

## Abstract

Publication bias refers to a systematic deviation from the truth in the results of a meta-analysis due to the higher likelihood for published studies to be included in meta-analyses than unpublished studies. Publication bias can lead to misleading recommendations for decision and policy making. In this education review, we introduce, explain, and provide solutions to the pervasive misuses and misinterpretations of publication bias that afflict evidence syntheses in sport and exercise medicine, with a focus on the commonly used funnel-plot based methods. Publication bias is more routinely assessed by visually inspecting funnel plot asymmetry, although it has been consistently deemed unreliable, leading to the development of statistical tests to assess publication bias. However, most statistical tests of publication bias (i) cannot rule out alternative explanations for funnel plot asymmetry (e.g., between-study heterogeneity, choice of metric, chance) and (ii) are grossly underpowered, even when using an arbitrary minimum threshold of ten or more studies. We performed a cross-sectional meta-research investigation of how publication bias was assessed in systematic reviews with meta-analyses published in the top two sport and exercise medicine journals throughout 2021. This analysis highlights that publication bias is frequently misused and misinterpreted, even in top tier journals. Because of conceptual and methodological problems when assessing and interpreting publication bias, preventive strategies (e.g., pre-registration, registered reports, disclosing protocol deviations, and reporting all study findings regardless of direction or magnitude) offer the best and most efficient solution to mitigate the misuse and misinterpretation of publication bias. Because true publication bias is very difficult to determine, we recommend that future publications use the term “risk of publication bias”.

**Supplementary Information:**

The online version contains supplementary material available at 10.1007/s40279-023-01927-9.

## Key Points

**Table:** 

Traditionally, assessing publication bias has been recommended for meta-analyses and as part of judgments concerning certainty of evidence (e.g., Grading of Recommendations, Assessment, Development, and Evaluations [GRADE]).
Funnel-plot based tests attempting to assess publication bias (and related concepts such as reporting bias and small-study bias) are underpowered and, furthermore, allow for multiple interpretations, and therefore publication bias cannot be ascertained.
At a minimum, “risk of publication bias” should be used instead of “publication bias,” and the inclusion and interpretation of this item in meta-analyses and in GRADE should be critically reviewed.

## Introduction

Systematic reviews containing meta-analyses descriptively combine and quantitatively aggregate the results of several individual studies (if sufficiently homogeneous) to summarize bodies of research evidence [1–11]. However, the results of systematic reviews can be biased when there is selective reporting of outcomes or statistical analyses within individual research studies depending on the direction, magnitude, and perhaps most profoundly, the statistical significance of their results [2]. Outcome reporting bias occurs when authors report only a subset, typically statistically significant, of all measured outcomes in the published article [4, 5, 12–23]. Reporting bias may be alternatively interpreted as an umbrella term encompassing several biases including publication bias [23–28].

Studies with statistically significant results are more likely to be published in peer-reviewed journals than studies with statistically non-significant results [3, 4, 9, 13, 14, 16, 17, 29–38]. Publication bias refers to the differential choice to publish studies depending on the nature and directionality of their results (Box 1) [3–5, 14, 16–18, 21, 23, 25, 29, 34, 38–42]. Authors regularly report study findings and submit for publication in peer-reviewed journals *only* when study findings are intriguing, impressive, and pass the threshold of statistical significance. Similarly, journal editors regularly accept submitted manuscripts for publication in peer-reviewed journals when study results will interest clinician and scientist readers, garner attention from media outlets, and enhance a journal’s Impact Factor—all of which are heavily influenced by whether study results are statistically significant [3–5, 14, 16–18, 21, 23, 25, 29, 34, 38–41].

This preferential publication of statistically significant studies regularly misrepresents the true distribution of individual study effect sizes and can cause misleading recommendations for decision making and informing policy [12, 39, 43]. Published studies, which usually have larger effect sizes than unpublished studies of the same sample size, are also more easily located in the scientific literature and thus more likely to be identified by and included in meta-analyses [2]. Although outcome or analytical (non-)reporting biases can be assessed if published studies have pre-registered/published protocols [4, 14–17, 19, 21, 26, 28, 43, 44], detecting publication bias is more complicated [14].

When the literature is dominated by small studies, as is common in the fields of sport and exercise medicine and sports science [45] (hereby referred to as sport and exercise science or SES), the results of meta-analyses are often contradicted by subsequent studies with larger sample sizes [30, 43]. Smaller studies tend to follow less rigorous methods and exhibit larger effect sizes [3, 5, 9, 27, 30, 43]. Regardless of the direction of the effects, the predominance of small studies, which experience greater effect size changes in response to systematic error (i.e., biases such as flexible analytical techniques) and random error (such as dramatic sampling variation), may bias the interpretation of meta-analytic results [26, 46]. Larger studies, despite not being immune from biases of their own that can negatively influence effect size accuracy [34, 42, 47, 48], identify a study effect size with greater precision (i.e., with smaller variability).

Assessing for potential publication bias in meta-analyses judges whether a summary effect size might be biased. There are many methods to assess the potential presence of publication bias in a meta-analysis. Both graphical plots and statistical tests exist to consider (i) whether there is any evidence of publication bias, (ii) whether the summary effect size in a meta-analysis might be at least partly due to publication bias, and (iii) what influence publication bias might yield on the summary effect size [49]. This educational review aims to educate the SES research communities on methods to assess potential publication bias in meta-analyses, their use, and how the interpretation of assessment findings can influence review conclusions. The focus is on funnel-plot based tests, owing to their prevalence in systematic reviews [42].

**Table:** 

**Box 1. What is Publication Bias?**
Statistically significant studies are more likely to be published, are more easily identified in the scientific literature, and are thus more likely to appear in meta-analyses compared with statistically non-significant studies. Because studies with larger effect sizes are more likely to be statistically significant than studies with smaller effect sizes (given the same sample size), studies included in meta-analyses tend to have systematically larger effect sizes than those that are not identified for inclusion in meta-analyses. Hence, publication bias refers to a systematic deviation from the truth in the results of a meta-analysis due to the higher likelihood for published studies to be included in meta-analyses than unpublished studies. Assessing publication bias using visual plots or associated statistical tests cannot conclusively determine whether included study effect sizes and potential “missing” studies overestimate the true summary effect size in a meta-analysis. Therefore, ‘risk of publication bias’ or ‘potential publication bias’—rather than simply ‘publication bias’—is what is being assessed to judge the risk on whether publication bias is present.

## Is There Possible Evidence of Publication Bias: What Does Funnel Plot Asymmetry Really Mean?

The presence of potential publication bias in a meta-analysis is usually assessed by analyzing funnel plot asymmetry [1, 2, 4, 5, 8, 9, 12, 18, 27, 29, 30, 39, 40, 43, 50, 51]. Funnel plots are scatterplots [5, 8, 12, 25, 27, 29, 52] that plot some measure of study size (e.g., sample size or standard error) against a measure of study effect size [1, 4, 9, 29, 30, 40, 43, 53]. Several metrics can be used to represent study effect size, such as standardized binary effect size metrics (e.g., odds ratio or risk ratio) [10, 12] or standardized continuous effect size metrics (e.g., Hedges’ *g*, partial eta-squared). The estimated precision of a study’s effect size increases as the study sample size increases [29, 30, 43]. Funnel plots may be contour enhanced, presenting one or more areas of statistical significance (e.g., *p* < 0.1, *p* < 0.05, and/or *p* < 0.01 with the 90%, 95%, and/or 99% confidence intervals, respectively), and are particularly useful to identify outliers and guide sensitivity analyses [5, 17, 35, 40, 50, 53]. Funnel plots can also display a second superimposed vertical line that signals the null effect size (e.g., ln odds ratio = 1 or Hedges’ *g* =  ~ 0) to easily identify studies with an effect size close to the null effect, which are likely to be non-significant [27].

Smaller studies produce effect sizes that vary widely, have larger standard errors, and are dispersed near the base of the funnel plot. In contrast, larger studies produce more consistent effect sizes and have a narrower distribution of effect sizes near the top of the funnel plot. This results in a smaller standard error and an inverted funnel shape [4, 5, 9, 12, 17, 24, 27, 29, 30, 39, 43, 50, 53]. Funnel plots can be created with a weighted analysis to reduce small-study effects (i.e., smaller studies have more extreme effect sizes than larger studies) [31, 43]. However, larger studies with fewer outcome events will be less powerful than small studies with more outcome events so studies with distinct sample sizes can present similar standard errors [10].

**Fig. 1:**
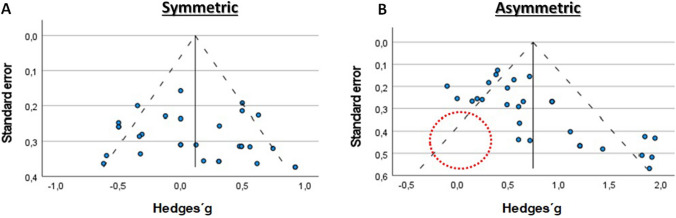
Illustration of a visual inspection of a funnel plot to assess the risk of publication bias. The blue dots represent individual studies. **A** A symmetrical funnel plot usually interpreted as reflecting an absence of publication bias. **B** An asymmetrical funnel plot typically interpreted as reflecting the presence of publication bias. The red dotted circumference denotes an empty space where studies were expected to be present

Visually inspecting funnel plot asymmetry (Fig. 1) is highly subjective and potentially misleading [5, 17, 30, 39, 43, 46, 51, 52] and, in practice, funnel plot-based methods are commonly interpreted as referring to publication bias instead of denoting small-study effects [42]. When 41 medical researchers visually observed funnel plots with ten studies each, accurate funnel plot asymmetry was identified for only 52.5% of plots [32]. Many statistical methods have been devised to complement funnel plot interpretation (Box 2). Although some of these tests (e.g., Egger’s regression) are more prone to assessing small-study effects [43], others (e.g., a robust Bayesian meta-analysis) attempt to assess a broader concept of publication bias [38]. Choosing the most appropriate method depends on assumptions that are often unverified by reviewers [52], and there is an ongoing debate over which tests and metrics should be used to generate the funnel plot [8, 10, 17, 18, 22–24, 33, 36, 40, 52]. Moreover, funnel plot interpretation is prone to “cherry-picking” [38] and statistical tests are each associated with different limitations. A thorough discussion of each test and the corresponding limitations falls outside the scope of our review.

**Fig. 2:**
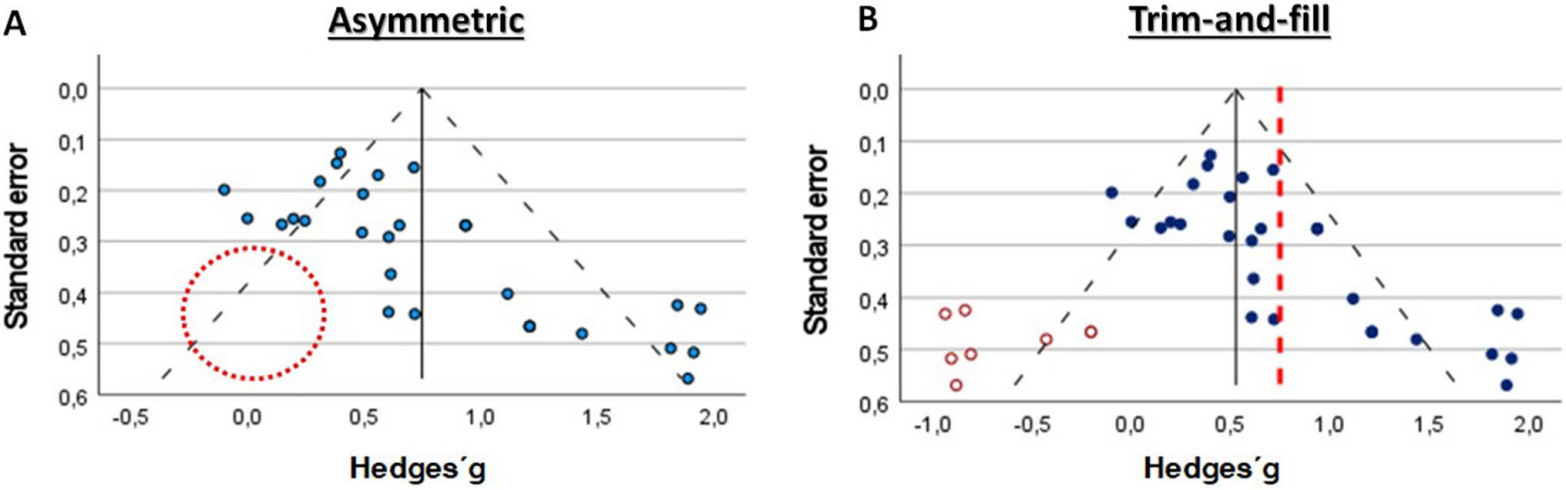
Illustration of the Trim-and-Fill statistical test to correct the funnel plot for publication bias. The *blue dots* represent individual studies. **A** Absence of studies on the left side of the funnel plot (red dotted circle). **B** Trim-and-Fill correction by adding imputed studies (red circles) and correcting the pooled Hedge’s g (previous pooled effect sized in the red dotted line)

A common misconception is that funnel plots will be symmetrical when publication bias is absent and asymmetrical when publication bias is present [5, 8, 9, 29, 30, 39, 43]. For example, smaller (and potentially underpowered) studies with statistically non-significant results are likely to remain unpublished, resulting in an asymmetrical funnel plot [17, 30]. When funnel plot asymmetry is visible, statistical methods aim to provide insight into how potential publication bias may influence the summary effect size. For example, the Trim-and-Fill method (Fig. 2) “balances” the funnel plot by re-iteratively removing the most extreme small studies from the positive side of the funnel plot, which generates a less biased summary effect size. To ensure a valid estimate of precision for the summary effect size, “trimmed” effect sizes are returned to the plot alongside imputed missing study effect sizes in the lower “statistically non-significant” quadrant of the funnel plot [24, 35, 39, 50, 66]. This procedure is problematic because the imputation of new “missing” effect sizes represents a daring assumption that such studies with those effect sizes might exist [34] and should be reserved only for sensitivity analyses [35, 39]. Indeed, it has been repeatedly demonstrated that if a funnel plot is asymmetrical and skewed, publication bias *may* be present [29], but it cannot be considered conclusive evidence of publication bias [17, 30, 51].

There are alternative (and often co-existing) sources of funnel plot asymmetry such as between-study variation (i.e., true heterogeneity), choice of effect size metric,^1^ statistical artefacts, chance, and even fraud [1, 4, 5, 8, 10, 12, 16–18, 21, 23–25, 27, 30–33, 35, 36, 40, 42, 43, 47, 48, 50–52, 68]. A common source of true heterogeneity emerges from the type of samples in included studies, especially when studying patients at a higher risk of an outcome: smaller studies may focus on this subset of the population, while larger studies may include a more diverse sample [9, 26, 30]. Small studies produce results that differ systematically (e.g., by having larger effect sizes) from results produced by larger studies (with smaller effect sizes), generating perceived funnel plot asymmetry [10, 26, 52]. Sensitivity analyses, subgroup analyses, and meta-regression models may be used to explain between-study heterogeneity [2, 3, 5, 8, 11, 17, 30, 42, 46, 69]. Similarly, network meta-regression can be implemented to assess small-study bias in a network meta-analysis [14]. Despite the availability and use of these techniques, many sources of true heterogeneity remain unknown [27, 32].

Any estimate of precision is subject to (i) random error due to sampling variation [23, 25, 31, 40, 70], (ii) the method of outcome aggregation (e.g., continuous versus binary) [71], (iii) the choice of specific outcome metric (e.g., risk ratio vs risk difference) [71], and (iv) the funnel plot axis, which combined or in isolation may explain funnel plot asymmetry [8, 10, 17, 24, 33, 36, 43, 52, 70]. For example, the method of outcome aggregation can have a relevant effect because continuous outcomes tend to display high levels of true heterogeneity [2, 33]. The true underlying effect size in the population, the distribution of study sample sizes, and whether one-tailed or two-tailed tests are employed can influence funnel plot asymmetry [23, 31].

In a review of 198 published meta-analyses that examined changes in the findings of funnel plots based on definitions of precision (e.g., standard error or sample size) and choices of the outcome metric (e.g., risk difference and relative risk), Tang and Liu [1] identified three general scenarios: (i) funnel plots that were always symmetrical; (ii) funnel plots that were always asymmetrical (although in some cases the *direction* of the asymmetry changed); and (iii) funnel plots that could be either symmetrical or asymmetrical depending on the definition of precision used and the outcome metric selected. As there is no consensus on which definitions of precision and outcome metrics should be used, interpreting funnel plot asymmetry warrants caution [1].

**Table:** 

**Box 2. Examples of Plots and Statistical Tests to Assess the Risk of Publication Bias**
*Is there possible evidence of publication bias?*
Forest plots.
Funnel plots.
Doi plot [54].
*What is the degree of funnel plot (a)symmetry?*
Egger’s weighted regression [43].
Begg’s and Mazumdar’s rank correlation test [55].
Macaskill’s funnel plot regression [31].
Peters’ regression test [56].
Debray’s regression using estimates of asymptotic precision as study weights [46].
Luis Furuya-Kanamori index [54].
Meta-regression residuals and inverse sample size [33].
Imbalance and asymmetry distance [51].
*Is the summary effect size at least partly due to publication bias?*
Rosenthal’s and Orwin’s Fail-safe N [37].
*How much might publication bias influence the summary effect size?*
Duval and Tweedie’s Trim-and-Fill method [39].
Copas’ selection model [3].
Precision-Effect Test and Precision-Effect Estimate with Standard Errors [22].
Rücker’s Limit Meta-Analysis Method [57].
*Is the p-value distribution affecting the meta-analysis a true effect and how large is this effect?*
p-curve [58–60].
p-uniform method [61, 62].
*Are p-values affecting the probability of selection of study for publication and how can it affect the meta-analysis true effect?*
Three-parameter selection model [61, 62].
Fixed Weights Selection Model [61–63].
*Is there evidence bias from selective (non)inclusion of results?*
Potential bias index [64, 65]
Risk Of Bias due to Missing Evidence tool [2]
*How much might both small-study effects and selection for statistical significance influence the summary effect size?*
Robust Bayesian Meta-Analysis [38].

## Are Current Methods Powerless?

Meta-analyses often include a limited number of studies, and many of these included studies possess small sample sizes. Consequently, statistical tests used to detect the risk of publication bias are frequently underpowered [5, 9, 18, 23, 25, 27, 31, 32, 38, 43, 46, 51, 52], although not all tests are equally affected. Statistical tests such as the Begg’s and Mazumdar’s rank correlation test [55] assess the risk of publication bias and regularly apply a statistical significance threshold of 10% (*α* = 0.1) with 90% confidence intervals [1, 9, 30, 43]. With *p* < 0.1, a false-positive rate of 10% may be attributed to chance alone [72]. False-positive findings also arise in the presence of large treatment effects, few events per study, or when all studies have similar sample sizes [30], and can occur regardless of the definition of precision or choice of effect measure [1]. However, the effect measure (e.g., binary vs non-binary) and the specific test applied may result in different false-positive rates. For example, the commonly used Egger’s regression test and Begg’s rank correlation test may be prone to false positives, especially when investigating binary outcomes [67].

However, common tests that assess the risk of publication bias (e.g., Egger’s regression test [43], Precision-Effect Test and Precision-Effect Estimate with Standard Errors [23]) have low statistical power to detect the risk of publication bias even when it is truly present [12, 27, 39, 55]. This means there may be publication bias despite symmetrical distribution [25, 27, 32, 36, 51, 52], and this has been observed even when 21 studies were available [31], which is above the usual *arbitrary* threshold of ten studies that is deemed necessary to exceed in order to assess for potential publication bias [2, 5, 14, 25, 27, 35, 51, 52]. When substantial heterogeneity is present, this number should be considerably greater than ten studies [27, 51].

Simulation analyses of rank-based and regression-based methods to assess the risk of publication bias in meta-analyses demonstrated that both methods have reduced statistical power, especially when including fewer than ten [30], or even ~ 20, studies [31]. Proponents of the rank correlation test, which assesses the association between study standard errors and effect estimates, proposed that this method requires a minimum of 25 studies to achieve moderate statistical power [55]. However, other authors have recommended at least 30 studies to achieve moderate statistical power [52]. Simulation studies further suggest that even when including more than 50 studies, analyses can be underpowered [46, 51]. When effect sizes are small, such statistical tests can remain underpowered even when as many as 63 studies are available [31]. Despite these test-specific limitations relating to statistical power, meta-analysts regularly interpret funnel plots containing fewer than ten studies [32, 51, 52], often at the request of uninformed reviewers. Although statistical tests are superior to visual inspection of funnel plots when assessing the risk of publication bias, they are underpowered given the number of studies commonly included in meta-analyses [31].

## How is the Risk of Publication Bias Being Interpreted in SES?

It is largely unknown how the risk of publication bias is assessed, whether through visually inspecting funnel plots or interpreting relevant associated statistical tests, within meta-analyses published in SES. Therefore, in mid-May 2022, we extracted all systematic reviews containing at least one meta-analysis published in 2021 in the *British Journal of Sports Medicine* and in *Sports Medicine* (i.e., the SES journals with the highest impact factors of 13.800 and 11.136, respectively, at the time of searching). We initially searched systematic reviews through PubMed^2^ and double-checked with direct searches on each journal’s website. This meta-research section should be viewed as an illustrative example and not as a formal and complete systematic review in the topic.

We identified 75 systematic reviews with at least one meta-analysis (24 in the *British Journal of Sports Medicine* and 51 in *Sports Medicine*; a complete list is provided in the Electronic Supplementary Material). Of these, 24 reviews (32.0%) did not address the risk of publication bias. In these reviews, it was unclear whether the authors had no intention to assess the risk of publication bias, if assessing the risk of publication bias was impossible because of between-study heterogeneity, or if there was an insufficient number of included studies and the review authors decided to overlook reporting this component in the article.

The remaining 51 systematic reviews reported an intention to assess the risk of publication bias. Sixteen of these systematic reviews (31.4%) used conservative terms such as variations of “potential/risk of publication bias” (*k* = 11), “risk of bias” (*k* = 2), “risk of small study bias/potential small study effects” (*k* = 2), or simply objective terms such as “funnel plot asymmetry” (*k* = 1). Five reviews applied the relatively conservative term “small-study effects,” and one review used the less conservative term “small-study bias” (k = 1). However, most systematic reviews (62.7%), including one of the reviews using the term “small-study effects”, used the more definitive term “publication bias” (*k* = 32). Of note, five reviews applied more than one expression (e.g., “small-study effects” plus “publication bias”). Using more cautious and conservative language involving the words “risk,” “potential,” or “expected” is recommended, but this was only verified in ~ 40% of the reviews.

**Fig. 3:**
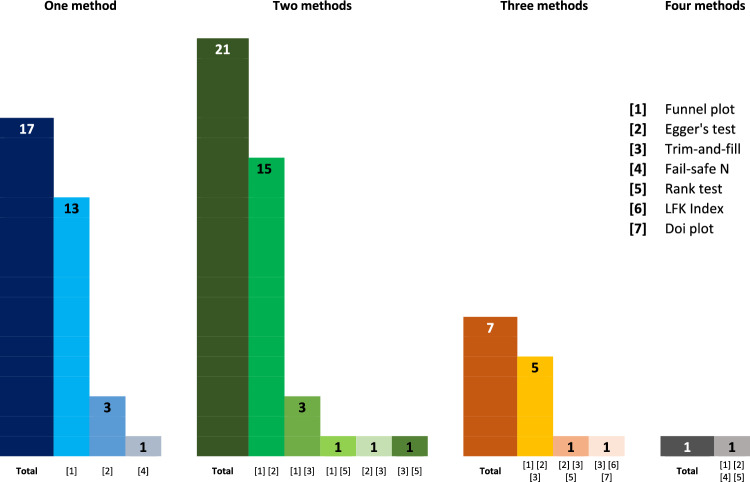
Summary of the number of methods used to assess the risk of publication bias and the most used tests. *LFK* Luis Furuya-Kanamori

Of 51 systematic reviews that planned to assess the risk of publication bias, five reviews did not perform this assessment because of a perceived insufficient number of included studies. Therefore, our analysis focuses on the remaining 46 systematic reviews containing at least one meta-analysis. Systematic reviews frequently used one (37.0%, *k* = 17) or two (45.7%, *k* = 21) method(s) to assess the risk of publication bias (Fig. 3). Visually inspecting funnel plots alone (without any statistical test) was performed to assess the risk of publication bias in 13 of the 46 systematic reviews (28.3%) — this is particularly worrisome given the aforementioned limitations of such an isolated approach. Egger’s test was the most applied statistical test (56.5%, *k* = 26), whether in isolation (6.5%, *k* = 3), combined with other statistical tests (17.4%, *k* = 8, most commonly with Trim-and-Fill), or most often in addition to visual inspection of funnel plots (32.6%, *k* = 15). The Trim-and-Fill method complemented other methods in 12 systematic reviews (26.1%). Other statistical tests were less frequently used.

Only 12 of the 41 systematic reviews (26.1%) reported that a minimum of ten studies was required to assess the risk of publication bias. Two of these systematic reviews stipulated this criterion for Egger’s test but not for visual inspection of funnel plots (Table 1**).** Although approximately half of the systematic reviews (52.2%, *k* = 24) assessed the risk of publication bias using the “10-study minimum” rule of thumb, 14 systematic reviews (30.4%) did not define a minimum number of studies to assess the risk of publication bias. Eighteen systematic reviews (39.1%) assessed the risk of publication bias even when fewer than ten studies were included in each meta-analysis. Two systematic reviews (4.3%) did not apply a statistical test but interpreted funnel plots with fewer than ten studies, and two systematic reviews did not report sufficient information to determine how the risk of publication bias was assessed. Of the 46 systematic reviews published in 2021 in the then two highest-ranked journals in the Sports Sciences section of Thomson Reuters Journal Citation Reports that addressed the risk of publication bias, nearly half (47.8%, *k* = 22) assessed for the risk of publication bias when fewer than ten studies were included in eligible meta-analyses, which contradicts existing recommendations [2, 5, 14, 25, 27, 30, 35, 43, 51, 52].

**Table 1:** Summary of studies assessing the risk of publication bias by pre-defining and using the “10-study minimum” heuristic

The “10-study minimum” heuristic	*k* (%)
Pre-defined a minimum of 10 studies?	
Yes	10 (21.7)
No	34 (73.9)
Only for Egger’s test	2 (4.3)
Implemented the criterion of ≥ 10 studies?	
Yes	24 (52.2)
No	18 (39.1)
Only for Egger’s test	2 (4.3)
Unclear	2 (4.3)

Overall, systematic review authors overlooked methodological concerns about the potential risk of publication bias, even when considering only recent systematic reviews (containing at least one meta-analysis) published in the two highest Impact Factor SES journals at the time. The results of the descriptive meta-research study performed within this education review suggest a misuse of currently available methods to assess the risk of publication bias. This is in line with recent observations regarding common errors in meta-analyses in the field [73]. Greater efforts should be implemented to properly educate researchers about when and how to assess the risk of publication bias reliably.

## Alternatives to Funnel Plots and Associated Statistical Tests: Can We Truly Assess the Risk of Publication Bias?

When meta-analyses report on the risk of publication bias, small-study bias is commonly being assessed [8, 17, 33, 35, 46]. Assessments of small-study effects require at least ten studies with varied sample sizes (and at least a medium pooled sample size) [24, 27, 30, 31, 33, 52]. Even the term “small-study bias” can be misleading because heterogeneity (e.g., in interventions or sub-populations) may explain funnel plot asymmetry [32]. In summary, funnel plot asymmetry is not necessarily a measure of the risk of publication bias; using it for that sole purpose can lead to inaccurate conclusions about the presence or absence of publication bias [17, 27, 32, 34, 51, 52]. Therefore, can we assess the risk of publication bias in meta-analyses without relying on funnel plot asymmetry?

Not all statistical methods designed to assess the risk of publication bias are built the same. For example, selection models tend to perform well under a variety of distributions and could be a valid alternative to funnel-plot based reporting practices, as they do not rely on the assumptions criticized in this review [42]. However, these models present their own limitations (cf., [3, 42, 63, 69]). Even proponents of selection models state that “correcting for this bias is not possible without making untestable assumptions” [3] (p. 247), or that “the author of the meta-analysis, then, is faced with a logically impossible task: to show that publication bias is not a problem for the particular data set at hand” [63] (p. 438). Perhaps quantitative methods should be complemented with qualitative analyses.

The Cochrane Bias Methods group has developed instruments that aim to assess bias due to missing evidence in a meta-analysis [2, 64] and a network meta-analysis [14]. The Risk of Bias due to Missing Evidence (ROB-ME) tool first assesses the risk of reporting bias by confronting published papers with pre-registered protocols that are included in systematic reviews, and then infers the risk of publication bias by scrutinizing systematic reviews’ search strategy and patterns of reported results [2, 64]. The Risk of Bias due to Missing Evidence in Network meta-analysis (ROB-MEN) tool [14] starts by assessing within-study bias due to unavailable results (i.e., [non-]reporting bias); then, it assesses between-study bias due to unpublished studies (i.e., publication bias). Like instruments that appraise the risk of bias in individual studies, ROB-ME and ROB-MEN tools openly involve rulers’ subjective personal judgments [2, 14, 64], albeit using standardized and objective criteria to inform such judgments.

Key outcomes or treatment comparisons are regularly expected in research on a specific injury or condition [11, 17, 20]. For example, in rehabilitation from sports injuries, time to return to play, and rate of reinjuries are commonly reported. However, it is possible that because of their informational value, authors of original studies included in systematic reviews were not interested in a subset of outcomes and therefore did not analyze them. In the previously mentioned example, not all injury rehabilitation studies report the reinjury rate. Likewise, there may be no consensus about what outcomes that should be prioritized in other research areas. For example, in the fields of SES, there is currently no consensus on how to assess fitness in children and adolescents [74, 75] or return-to-sport after a lateral ankle sprain, respectively [76].

ROB-MEN also assesses a *suspected* risk of publication bias in a network meta-analysis based on (i) failure to search gray literature or track study registrations for unpublished studies, (ii) novelty of a research field/topic, and (iii) previous evidence of publication bias for a given outcome or treatment comparison [14]. These assessments are complex, and the creators of ROB-MEN openly state that assessing the risk of reporting bias is more easily quantified than the risk of publication bias [14]. Indeed, “correcting for this bias is not possible without making *untestable* assumptions (p. 247)” [3], and publication bias cannot be definitively ruled out in most meta-analyses [4]. Conversely, publication bias cannot be conclusively ruled in either, as statements of missing studies are mere assumptions [24].

The certainty of the evidence of findings in a systematic review is most commonly assessed using the Grading of Recommendations, Assessment, Development, and Evaluations (GRADE) [2]. Using this framework, the certainty of the evidence is downgraded by one level upon suspected publication bias [34]. GRADE openly states that funnel plot asymmetry is not evidence of publication bias. GRADE advises users to suspect publication bias when the evidence derives from only a few studies and/or if most studies have underlying commercial interests [34]. Although we understand the relevance of such advice, the existence of few studies may reflect the novelty of the research field instead of indicating publication bias. As many meta-analyses contain fewer than ten studies, assessing the risk of publication bias is unreliable; thus, few systematic reviews perform this assessment. Considering the concerns associated with assessing the risk of publication bias, there is a risk (albeit small) of erroneously downgrading the certainty of evidence when publication bias is not present [33]. Based on the limitations outlined throughout our educational review and the often misused and misinterpreted risk of publication bias, we question the validity of the ‘publication bias’ GRADE domain in many of the published meta-analyses, especially when fewer than ten studies are available for the meta-analysis. Even in the presence of ten or more studies, interpretations should be carried out with a degree of caution.

## Not Discriminating Based on the Size of a Study’s *p*-Value: How Can We Reduce the Risk of Publication Bias?

Assessing “true” publication bias is frequently not possible but a few simple solutions can aid in mitigating the risk of publication bias.

### For Authors of Systematic Reviews:

(i) Pre-registration: pre-register your systematic review and ensure that the review protocol is detailed. Later, in the published manuscript, disclose all deviations from the pre-registered protocol and the reasons for these alterations.
(ii) Search strategy restrictions on date: avoid date limits in search strategy filters, except in well-justified cases (e.g., if the intervention of interest did not exist before a certain date).
(iii) Search strategy restrictions on language: avoid limiting a search by language of publication, even if most published papers on a topic are in English. Current automated translation technologies and the accessibility of native human translators mean that restricting literature searches by the language of publication is less necessary and justifiable.
(iv) Consider many shades of gray: gray literature^3^ (i.e., conference proceedings, PhD theses, pharmaceutical study reports) should be consulted in addition to published peer-reviewed literature [77]. Although some may argue that more reliable conclusions would be derived from peer-reviewed randomized studies, moderator analyses can be considered to compare between data derived from gray and non-gray literature.
(v) Corresponding authors as information sources: when a study lacks summary data/information that are required for inclusion in a systematic review, reviewers should contact the corresponding authors of the study to obtain the missing information/summary data before deciding whether to exclude the study from the meta-analysis. When data are presented only in figures, free software packages allow reliable extraction of relevant data, such as WebPlotDigitizer [78] (https://automeris.io/WebPlotDigitizer/).
(vi) An ongoing story: living reviews continually identify and incorporate new evidence at regular intervals and can provide an ongoing interpretation of a body of evidence. By regularly updating literature searches, living reviews can circumvent problems such as the time-lag bias [5, 17, 24, 25]. However, living reviews require platforms where updates can be easily uploaded and made available. Currently, the most practical solution is publishing the initial version in a peer-reviewed journal and providing links to open-access websites hosting future updates (e.g., Open Science Framework at https://osf.io).

### For Authors of Original Studies:

(vii) Pre-registration: pre-register/publish a study protocol before data collection. This allows systematic reviewers to identify the protocols of subsequently published studies and the protocols of studies that have yet to be published despite a sufficient timeline to allow for publication. This identification provides transparency to the conduct of the study (discouraging the authors from selective reporting) and can allow for a more reliable assessment of potential publication bias. Identifying study protocols also allows review authors to compare included publications with their pre-registered protocols (to assess and detect reporting bias) [68]. Some journals publish these works, with the title often including “study protocol for …” (e.g., [79]). Such reports are peer reviewed, which will help improve the rationale and methods even before data collection begins. The final paper should later be published regardless of the results, assuming the proposed data collection and analysis plan was followed. In any case, authors should explicitly state discrepancies between the registered protocol and the final published manuscript, and any explanations for those deviations.
(viii) Report everything: report all study findings (including statistically non-significant results). If the manuscript is too long, consider providing additional information in supplementary material or a link to external open-access platforms (e.g., Open Science Framework).
(ix) Not everything needs to be novel: there is considerable value in certainty from replication: perform replication studies, which help to better assess how robust certain observed effects are (i.e., if they are reproducible and replicable).
(x) Sharing is caring: consider making original individual participant data available to systematic reviewers and other researchers to increase transparency and allow data sharing for inclusion in an individual participant data meta-analysis.

## Concluding Remarks

The risk of publication bias may arise from many contributing factors and can often be challenging to evaluate and interpret. Here, we explored the case of funnel plot-based tests and how they could be misleading. Many researchers, including those within the SES field, are prone to (i) misunderstanding the concept of publication bias in meta-analyses (i.e., using definitive instead provisional statements), (ii) misusing funnel plots and associated statistical tests to assess potential publication bias, and (iii) misinterpreting subsequent results. However, it is possible to optimize meta-analytic methods and refine user interpretations to conduct better research that aids clinical decision and policy making. Funnel plot asymmetry should not be conflated with publication bias because (i) there can be publication bias despite a symmetrical funnel plot, (ii) there can be no publication bias despite an asymmetrical funnel plot, (iii) most existing statistical tests that assess the risk of publication bias are underpowered, and (iv) even the minimum threshold of ten studies to assess the risk of publication bias can be insufficient.

There are many methods to assess the risk of publication bias but ultimately all involve considerable subjectivity. Even when suspecting a high risk of publication bias (e.g., upon substantial funnel plot asymmetry from a meta-analysis containing many studies), it might be premature to dismiss the results of sound methodological studies [48, 52]. As discussed, alternative methods for addressing publication bias in meta-analyses—selection models and qualitative approaches—do not rely on the assumptions discussed in this educational review. Both researchers and readers should refrain from conclusively stating that there is, or there is not, publication bias. In line with previous recommendations [4], we endorse that systematic review authors should dilute stronger statements about the risk of publication bias, as they can be misleading. Based on the limitations outlined in our education review, we also recommend eschewing the GRADE domain of “publication bias” when judging the certainty of the evidence, especially when fewer than ten studies are available for the meta-analysis.

We encourage authors of systematic reviews to strive for the most rigorous methodological standards of systematic review conduct—pre-registration, avoid restricting search strategies by time and language, inspecting gray literature, contacting corresponding authors for additional study-level data, and performing living systematic reviews—to enable the most reliable results and recommendations for practice and policy. Similarly, authors of original research studies must consider open science principles—pre-registering study intentions before data collection, publishing registered protocols, reporting all outcomes, analyses, and results, performing replication studies, and making study data openly available for use by independent researchers. Only by harnessing open science principles when undertaking original research and including all relevant original research using rigorous systematic review methods, can publication bias and its burden be truly estimated. As key messages, we highlight that (i) “publication bias” should be replaced with “risk of publication bias” and that (ii) the commonly used funnel-plot based methods cannot rule out other sources of funnel plot asymmetry, and thus definite statements regarding the presence or absence of risk of publication bias should be avoided.

### Supplementary Information

Below is the link to the electronic supplementary material.

Supplementary file1 (XLSX 26 KB)

## Footnotes

1 There seems to be low agreement between different tests for publication bias (e.g., between Begg’s rank test and Egger’s regression test), and higher false-positive detection rates for binary outcomes [67].

2 Syntax for PubMed searches: *Sports Medicine*: (((“2021/01/01”[Date—Publication]: “2021/12/31”[Date—Publication])) AND (“Sports medicine (Auckland, N.Z.)”[Journal])) AND (review[Title] OR meta[Title]). *BJSM*: (((“2021/01/01”[Date—Publication]: “2021/12/31”[Date—Publication])) AND (“British journal of sports medicine”[Journal])) AND (review[Title] OR meta[Title]).

3 Abstracts usually focus solely on a few primary outcomes [16]. Therefore, if conference abstracts are included in the eligibility criteria, the reviewers should strive to contact the authors and obtain more complete information on study outcomes.

## Declarations

### Availability of Data and Material

The data used to inform the meta-review section are submitted as Electronic Supplementary Material.
